# Electrospun Coaxial Polycaprolactone/Polyvinylpyrrolidone Fibers Containing Cisplatin: A Potential Local Chemotherapy Delivery System for Cervical Cancer Treatment

**DOI:** 10.3390/polym17050637

**Published:** 2025-02-27

**Authors:** Mariana Sarai Silva-López, Vladimir Alonso Escobar-Barrios, Luz Eugenia Alcántara-Quintana

**Affiliations:** 1 Coordination for the Innovation and Application of Science and Technology (CIACYT), Autonomous University of San Luis Potosi, 550-2a Sierra Leona Ave, San Luis Potosí 78210, Mexico; marianasillop@gmail.com; 2 Advanced Materials Department, Institute for Scientific and Technological Research of San Luis Potosi A.C. Road to San Jose Dam, Lomas 4a Section, San Luis Potosí 78216, Mexico; vladimir.escobar@ipicyt.edu.mx

**Keywords:** cervical cancer, biomaterials, coaxial electrospinning

## Abstract

Cisplatin, a frequently used chemotherapeutic for the treatment of cervical cancer, causes adverse effects that limit its use. Treatment with local therapy that limits toxicity remains a challenge. The aim of this study was to develop a local intravaginal cisplatin delivery system of polycaprolactone/polyvinylpyrrolidone sheath/core fibers by coaxial electrospinning. Physicochemical properties, degradation rate, mucoadhesion, release profile, and in vitro biosafety assays were characterized. Microscopy images confirmed the coaxial nature of the fibers and showed continuous morphology and diameters of 3–9 µm. The combination of polymers improved their mechanical properties. The contact angle < 85° indicated a hydrophilic surface, which would allow its dissolution in the vaginal environment. The release profile showed a rapid initial release followed by a slow and sustained release over eight days. The degradation test showed ~50% dissolution of the fibers on day 10. The adhesion of the fibrous device to the vaginal wall lasted for more than 15 days, which was sufficient time to allow the release of cisplatin. The biosafety tests showed great cytocompatibility and no hemolysis. The characteristics of the developed system open the possibility of its application as a localized therapy against cervical cancer, reducing adverse effects and improving the quality of life of patients.

## 1. Introduction

Cervical cancer is one of the leading cancers affecting women worldwide, with 604,000 new cases and more than 300,000 related deaths reported annually [1]. In recent years, an increase in both the incidence and mortality of this type of cancer has been observed among young women, even those under 40 years of age. In Latin America, 83,200 women are diagnosed, and 35,680 die from cervical cancer each year, with a high proportion (52%) of patients under 60 years of age [2].

To combat advanced local cervical cancer, a combination of radiotherapy and chemotherapy is used to increase the survival rate of patients and try to ensure that the tumor does not recur due to the existence of residual cancer cells [3,4]. However, chemotherapeutic agents lack selectivity, causing cytotoxic effects on both cancer cells and normal cells, which causes severe adverse effects that may be transient or persist for long periods, such as thrombocytopenia, neutropenia, nephrotoxicity, neurotoxicity, anemia due to hematological toxicity, and bone marrow depression, which decreases the quality of life of patients [5]. This obstacle in treatment can be overcome by targeted or localized therapy, which limits the toxicity of chemotherapeutic agents by promoting their action at the tumor site, avoiding systemic action. This type of therapy offers advantages such as protection from drug degradation in the bloodstream, improved drug stability and bioavailability, selective administration, and decreased toxic side effects.

Easy access to the cervix through the vagina provides a convenient and suitable route for the local administration of drugs, such as chemotherapeutic agents. Currently, various devices are used for vaginal administration of drugs, such as vaginal tablets for the treatment of bacterial infections, as well as gels and films; however, these drug delivery vehicles have disadvantages, such as low intravaginal adherence and short residence time or low loading capacity of the drug in the pharmaceutical form [6,7,8].

Drug delivery systems made of natural or synthetic biomaterials, such as drug-loaded electrospun polymeric fibers, are being used at the micro- and nanometric scales. These systems offer significant advantages, such as high loading capacity due to the large surface area about the volume of the fibers, prolonged and controlled drug release, flexibility, biodegradability and biocompatibility, since the polymers that make up the fibers are compatible with the human body [9].

Coaxial electrospinning has been used to fabricate micro/nanofibers with sheath/core structures of different polymers in a single step, thereby achieving improved combined properties in a single fiber [10,11]. Electrospinning is a process in which fibers are obtained by stretching a viscoelastic polymer solution under an applied specific voltage, causing fine jets of solution to be expelled from the needle toward a collector, where the formed fibers are deposited [12].

Polycaprolactone (PCL) is an FDA-approved polymer of great importance for its mechanical properties, miscibility with a wide range of other polymers, thermal and chemical stability, permeability, biodegradability and biocompatibility. It also provides good mechanical rigidity at the physiological level [10,13]. Despite having applications in drug delivery systems, its use is limited by its hydrophobic nature, low encapsulation efficiency, and tendency toward unwanted burst release of the drug. However, these issues can be mitigated by using it in drug delivery systems in combination with other hydrophilic polymers such as polyvinylpyrrolidone (PVP). This synthetic polymer has applications as a drug carrier due to its hydrophilic nature, physiological compatibility, non-toxicity, temperature resistance, and pH stability [14,15,16].

The current study developed polycaprolactone/polyvinylpyrrolidone sheath/core coaxial fibers loaded with the chemotherapeutic drug cisplatin by coaxial electrospinning technique. The two polymers used were chosen for their biodegradability and compatibility with the human body; PCL, used for the sheath of the fibers, provides robust mechanical properties. In contrast, PVP was used for the internal part of the fiber due to its hydrophilic nature that favors the wettability of the fibers and their dissolution in the vaginal aqueous environment and release of cisplatin. Furthermore, PVP has adhesive properties that improve the intravaginal residence time. The chemotherapeutic agent used, cisplatin, encapsulated in the internal/nuclear part of the fiber, is the primary drug used in chemotherapy due to its high efficacy in cases of advanced cancer; however, it causes nephrotoxicity, which often necessitates its replacement by other chemotherapeutics. Delivering cisplatin by local application of electrospun fibers to the cervix could reduce adverse effects, including kidney damage, allowing for its continued use. Furthermore, the developed pharmaceutical form is a relatively easy alternative to apply, similar to vehicles currently used at the cervical level, such as ovules with medication to control infections. The developed polymeric system allows the release of the drug in a constant and sustained manner controlled by the degradation rate of the polymers, maintaining the drug’s bioavailability over extended periods. This study aimed to evaluate the fibers manufactured for their application in localized therapy for the treatment of cervical cancer. The goal was to reduce the side effects that the chemotherapeutic agent causes when applied systemically, which would improve patients’ quality of life during and after their healing process. To efficiently carry out the development of coaxial electrospun fibers, we first reviewed the reports from previous work on electrospinning of composite fibers of the polymers PCL and PVP that we used in this work, in addition to reviewing the influence of different parameters of the electrospinning process (strengths). Difficulties to overcome to obtain coaxial fibers were detected, such as the choice of electrospinning parameters that must be applied simultaneously to both polymer solutions, such as voltage (weaknesses). These observations highlighted the need to standardize two separate electrospinning processes for the individual fibers of each polymer before carrying out coaxial electrospinning (opportunities) could be clarified. Despite the inconveniences presented, such as the absence of needles of a specific diameter due to their use by other work equipment (threats), the development of coaxial PCL/PVP fibers was possible.

## 2. Materials and Methods

### 2.1. Materials

Polycaprolactone (PCL, Mn = 80,000 g/mol) and polyvinylpyrrolidone (PVP, Mn = 40,000 g/mol) were purchased from Sigma-Aldrich, Burlington, MA, USA. Chloroform and dimethylformamide (DMF) were obtained from J. T. Baker, Phillipsburg, NJ, USA. Cisplatin (CDDP) was supplied by Accord Farma, Mexico City, Mexico. All other reagents were used as received without further purification. The HeLa and NIH/3T3 cell lines were obtained from ATCC, American Type Culture Collection, Manassas, VA, USA (Chen TR. Re-evaluation of HeLa, HeLa S3, and HEp-2 karyotypes. Cytogenet. Cell Genet. 48: 19–24, 1988). Female Wistar rats weighing 90 to 100 g were provided by the Bioterium of the Biosciences Center of the Faculty of Agronomy and Veterinary Medicine of the Autonomous University of San Luis Potosi (FAV-UASLP).

### 2.2. Electrospinning of Coaxial Fibers

**Figure 1:**
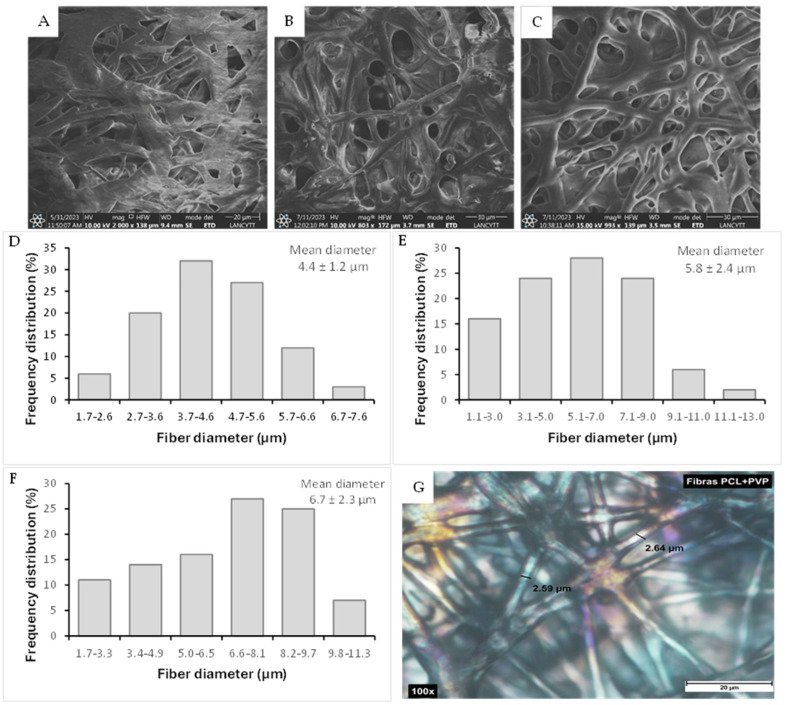
Scanning electron micrographs of fibers: (**A**) PCL/PVP fibers without drug; (**B**) PCL/PVP/CDDP 0.2 mg fibers; (**C**) PCL/PVP/CDDP 0.6 mg fibers. Histogram representation in (**D**–**F**) of average diameter distribution of fibers without drug and with drug (0.2 and 0.6 mg). (**G**) petrographic microscope images of fibers. Scale bars represent 30 µm.

To fabricate the coaxial fibers by electrospinning, individual polymer solutions of polycaprolactone and polyvinylpyrrolidone were initially prepared, for which the choice of suitable solvents for their dissolution and the concentration-viscosity of the resulting solution were considered, a factor that directly impacts the formation of the fibers. The coating solution was prepared by dissolving PCL (30% *w*/*v*) in a mixture of chloroform: DMF (3:1). PVP (85% *w*/*v*), the polymer of the inner part of the fibers, was dissolved in the same solvent mixture but in a different proportion (chloroform: DMF 2:1). The polymer solutions were kept under constant magnetic stirring for 24 h to achieve a homogeneous solution. To prepare the fibers, 0.2 and 0.6 mg of cisplatin were initially dissolved in 1 mL of DMF, based on previous reports of IC50 in SiHa, CaSki, C-33 A, and HeLa cells [11,17,18,19] and stirred for 12 h. The PVP solution was added to the above drug solution. The PCL/PVP sheath/core fiber was fabricated by a coaxial electrospinning system (Basic yet fully-equipped TL electrospinning & spray unit model TL-01, Tong Li Tech Co., Shenzhen, China). An experimental design was carried out to determine the optimal parameters, considering a review of previous reports and investigating the influence of process parameters on fiber formation; individual polymeric fibers were developed, and then coaxial electrospinning was carried out. The Supplementary Material provides additional information. Two individual syringes, connected via hoses in the concentric coaxial configuration, were used for the sheath and core solutions. The flow of polymer solutions was maintained using separate pumps, 3 mL/h and 2 mL/h for the sheath and core parts, respectively. The applied electric voltage, the distance between the needle and the collector, and the rotation speed of the latter were 10 cm, 13–16 kV, and 230–290 rps, respectively. The resulting randomly deposited fibers were collected on waxed paper. The fibers were left to dry in an oven to evaporate solvent residues. The fibers were observed with a Nikon Eclipse Ci POL (Metrolab S.A. de C.V., Mexico) petrographic microscope and NIS Element software D4.30.01 (Metrolab S.A. de C.V., Mexico) during the optimization of the electrospinning process, Figure 1G.

### 2.3. Physicochemical Characterization of the Fibers

#### 2.3.1. SEM Analysis

Morphological analysis of the fibrous device was obtained by observation in a Helios G4 CX dual-beam scanning electron microscope, (SEM) (microscope version 12.2, FEI company, Hillsboro, OR, USA) by mounting the samples on pieces of metal surface using double-sided conductive tape, using an acceleration voltage of 10 and 15 kV. Fiber diameters were analyzed with ImageJ software 1.54d (National Institutes of Health, Bethesda, MD, USA). The average fiber diameter and percentage size distribution were determined from the SEM images by measuring 100 fiber segments randomly selected from 5 fields. To confirm the coaxial nature of the fibers, cross-sectional micrographs of a fractured fragment of the sample previously treated with liquid nitrogen were obtained.

#### 2.3.2. FTIR Analysis

The presence of the polymer and drug functional groups in the coaxial fiber was determined by Attenuated Total Reflectance-Fourier Transform Infrared Spectroscopy analysis (ATR-FTIR) (Nicolet 6700 FT-IR, Thermo Fisher Scientific, Waltham, MA, USA) in the spectral region between 500 and 4000 cm^−1^. Spectra were obtained for each sample: pure PCL, pure PVP, cisplatin, PCL/PVP fibers, and PCL/PVP/cisplatin fibers.

#### 2.3.3. Contact Angle Analysis

To evaluate the hydrophobic/hydrophilic nature of the fabricated fibers, water contact angle measurements were performed using a goniometer using the sessile drop technique (Theta Lite Optical tensiometer, Biolin Scientific, Västra Frölunda, Sweden) and analyzed with OneAttension software V 1.8. A segment of coaxial fibers was placed on a glass slide. A drop of deionized water (2.5 µL) was placed on the surface of the fibers with a micrometer syringe; subsequently, images were captured to evaluate the contact angle using the Young—Laplace equation at 10 s time.

#### 2.3.4. Tensile Test

To evaluate the mechanical properties, the average tensile strength of the fibers was calculated using an Instron 3369 universal machine and Bluehill Lite Universal software V 2.35 (Instron, Barcelona, Spain); the test was performed at room temperature. The samples were cut to obtain a segment 10 cm long and 0.12 to 0.19 mm thick. The reference conditions for this test were a length of 100 mm and a speed of 1 mm/min. The force required for tearing was calculated as the tensile strength of the fibers. Four measurements were taken.

### 2.4. In Vitro Degradation Assay

The degradation of the fibers was determined by measuring their weight loss and FTIR analysis. Small fiber fragments (1 cm^2^) were cut and incubated in PBS pH 4.07 as a test medium at 37 °C for 10 and 14 days and 1, 3, and 6 months. After incubation, the fiber fragments were rinsed with water, air-dried, and weighed to determine their weight loss due to degradation. The weights of 3 independent samples were measured for each time point. In addition, FTIR spectra of the fiber fragments were obtained at the end of the test.

### 2.5. In Vitro Release Assay

Fragments weighing 20 mg of PCL/PVP/cisplatin fibers were immersed in 10 mL of PBS (pH 4.07) at 37 °C. At predetermined intervals, 1 mL of release buffer was removed for analysis and replaced with 1 mL of fresh PBS for continuous incubation. The amount of cisplatin present in the buffer was determined by UV-Vis spectroscopy at 202 nm (Thermo Fisher Scientific microplate photometer, Life Technologies Holdings PTE LTD, Singapore). The results were presented as a percentage of cumulative release (Equation (1)):

(1) $$Cisplatin\;release\;\left(\%\right)=\frac{Qt}{QT}\times 100$$

*Qt* is the amount of cisplatin released at time *t*, and *QT* is the total amount of cisplatin in the fibers. Samples were processed in triplicate. The calibration curve was performed based on standard solutions of cisplatin with concentrations ranging from 0.01 to 1.4 mg/mL.

### 2.6. Hemolysis Assay

Erythrocyte suspension was used, which was exposed to segments of the fibers. Incubation was carried out at 37 °C for 3 h with constant agitation. The samples were then centrifuged at 1400 rpm for 10 min, and the absorbance of hemoglobin released in the supernatant (1 mL) was recorded at 545 nm. NanodropOne (Thermo Fisher Scientific, Madison, WI, USA) was used to measure absorbance. The erythrocyte suspension with PBS was taken as a negative control, while the erythrocyte dilution with 20% Triton-X-100 was taken as a positive control.

The blank was 1x PBS. The assay was performed in triplicate. The percentage of hemolysis was calculated using the following equation (Equation (2)):

(2) $$Hemolysis\;\left(\%\right)=\frac{Asample-Anegative}{Apositive-Anegative}\times 100$$

### 2.7. Biocompatibility Assay

For this assay, NIH3T3 cells (mouse embryonic fibroblasts) were used. Cell viability was determined using the MTT method. The assay aimed to evaluate the biocompatibility of PCL/PVP fibers in non-cancerous cells. A disk of approximately 10 mg of PCL/PVP fibers (previously sterilized under UV light) was added to the wells (96-well plate) with medium and cells in triplicate, and they were incubated for 24 h. As a negative control, wells with cells in culture medium were used, and as a positive control, cells treated with a 10% hydrogen peroxide solution were used. After incubation, the culture medium and treatments were removed and 50 µL of MTT solution was added. The microplate was incubated at 37 °C, 5% CO_2_ for 4 h. After incubation, it was transferred to a microplate reader with a 570 nm filter to read the absorbance. Cell viability was calculated relative to cell viability under control conditions (Equation (3)):

(3) $$Viab.\%=\frac{100\times OD570e}{OD570b}$$

*OD*570*e* = is the mean value of the measured optical density of the test sample.
*OD*570*b* = is the mean value of the measured optical density of the blank.

### 2.8. Cytotoxicity Assay

HeLa cells (1 × 10^4^)/well were seeded with exposure to segments of electrospun coaxial fibers to evaluate their cytotoxic capacity and cultured for 72 h. A solution of 50 μL of MTT 3-(4,5-dimethylthiazol-2-yl)-2,5-diphenyl tetrazolium bromide) was added to each well and incubated at 37 °C for four hours. The absorbance was observed at 570 nm, as described in the previous paragraph.

### 2.9. Assessment of Mucoadhesion

Four female Wistar rats (~90–100 g) were used for this assay, from which the vaginal tissue was carefully isolated to preserve the mucosa; the protocol for its use in this assay was approved by the CIACYT Research Ethics Committee (registration number CIACYT-CEI-007). To humanely euthanize the rats, the procedure was carried out following the Mexican Official Standard NOM-062-ZOO-1999 [20], which dictates the technical specifications for the production, care, and use of laboratory animals to eliminate or minimize pain and stress. The animal was placed in a closed receptacle containing cotton soaked with chloroform, ensuring that the skin was not in direct contact with the anesthetic; inhalation of the vapors led to cessation of breathing and death. To determine the residence time of the fibers on the mucosa, a vaginal tissue sample was fixed to a glass slide with cyanoacrylate adhesive; the fibers fragment (1 cm^2^) was then adhered to the mucosa by applying a pressure of 500 g for 30 s. It was then placed at a 60° angle in a beaker with simulated vaginal fluid (SVF). The preparation was placed at 37 °C and agitated at 100 rpm. Residence time was determined by observing the samples in quadruplicate.

### 2.10. Statistical Analysis

Statistical analysis was performed using GraphPad Prism 10.4.1 software (Boston, MA, USA). Results were expressed as mean ± standard deviation. Unpaired Student t-tests and one-way ANOVA were performed when appropriate, with subsequent post hoc testing; a *p*-value < 0.05 was considered statistically significant.

## 3. Results and Discussion

### 3.1. Morphological Evaluation

SEM micrographs showed developed fibers with continuous morphology, without beads or interrupted segments, with a rough surface and random orientation, as shown in Figure 1. The concentration and viscosity of the polymer solution play an essential role in obtaining fibers, since an excessive increase in viscosity prevents the passage of the solution through the capillary in the electrospinning system; on the other hand, very low viscosity values generate breakage of the fibers in the form of drops [21]. The average diameters obtained by analysis with ImageJ software were compared between fibers without CDDP and fibers with this drug. The average diameter of coaxial fibers without the drug was 4.43 ± 1.18 µm; coaxial fibers PCL/PVP loaded with the drug presented an average diameter of 5.78 ± 2.41 µm and 6.72 ± 2.29 µm for loading 0.2 mg and 0.6 mg of cisplatin, respectively. According to the results obtained, the cisplatin fibers showed a slight increase in the average diameter, which could be attributed to the ionic nature of cisplatin. When cisplatin was added to the PVP nuclear solution for electrospinning, the voltage necessary to obtain the fibers increased, which consequently increased their diameter; that is, the composition of the polymer–drug solution affects parameters of the electrospinning process such as voltage, which produces variations in the size or morphology of the fibers [21,22]. During the electrospinning process, it is essential to standardize and control the different parameters that influence the manufacture of the fibers to obtain them uniformly, without drips, to avoid uneven loads of the drug to be encapsulated. However, once the electrospinning process is standardized, it is possible to produce the fibers in a simple way and on a large scale in a short time.

**Figure 2:**
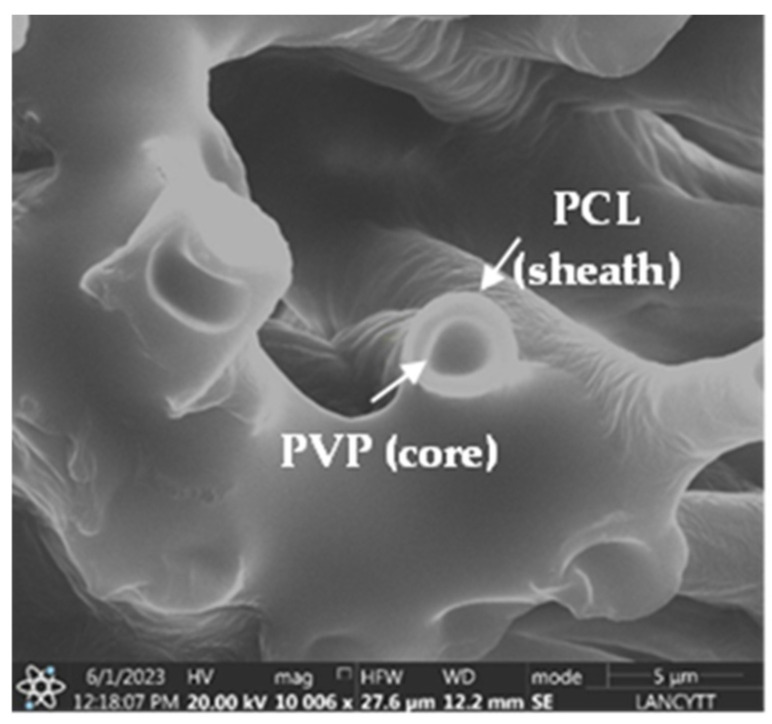
SEM micrograph of PCL/PVP coaxial fibers fabricated by electrospinning (conditions of electrospinning: 2 mL/h internal, 3 mL/h external flow, 10 cm distance between the needle and the collector, 13–16 kV applied voltage).

Furthermore, as shown in the SEM micrograph in Figure 2, a clear differentiation between the core and sheath layers of the fiber is observed, confirming the successful formation of the PCL/PVP coaxial fiber, as previous studies have reported [23,24]. These results determine that the CDDP drug was found encapsulated in the polymeric core part of the fiber. Moreover, the delimitation between the inner and outer layers of the fiber was achieved by a fast and efficient electrospinning process. Despite employing the same mixture of solvents in the preparation of the polymeric solutions, albeit in different proportions, the immiscibility of the polymeric solutions was achieved [25].

### 3.2. Functional Group Analysis

**Figure 3:**
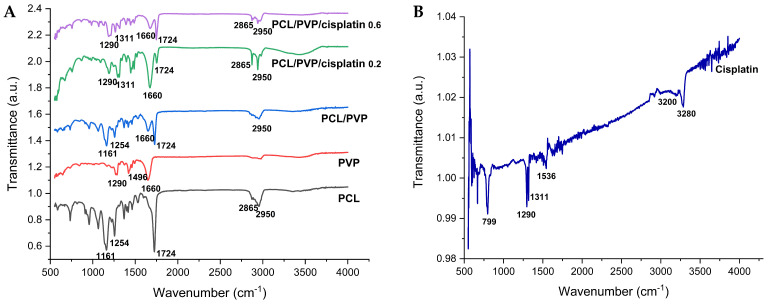
FTIR spectra of (**A**) pure components (PCL, PVP), electrospun coaxial fibers (PCL/PVP, PCL/PVP/CDDP), and (**B**) cisplatin.

FTIR spectroscopy was used to determine the presence of the biopolymers and cisplatin in the coaxial fibers. Figure 3 shows the transmittance spectra of PCL, PVP, cisplatin, and PCL/PVP fibers with and without the drug. Characteristic PCL bands are observed at 1161 cm^−1^ (C-O stretching), 1254 cm^−1^ (C-O-C stretching), 1724 cm^−1^ (C=O stretching), 2865 cm^−1^ (CH_2_ symmetric stretching), and 2950 cm^−1^ (C-H stretching). The bands 1290 cm^−1^ (CH_2_ twisting), 1496 cm^−1^ (CH_2_ scissor mode), and 1660 cm^−1^ (C=O stretching) belong to polyvinylpyrrolidone components [26,27]. Finally, the bands corresponding to cisplatin were 799 cm^−1^ (N-H stretching), 1290 cm^−1^ (symmetric amine bending mode), 3200 cm^−1^ (amine stretching), 3280 cm^−1^ (amine stretching), 1536 cm^−1^ (N-H stretching), and a prominent band of 1311 cm^−1^ (N-H stretching), which can be observed in the spectra of drug-loaded fibers [11]. The appearance of bands corresponding to polymers and cisplatin in the spectra demonstrates their presence in the manufactured fibers.

### 3.3. Contact Angle

**Figure 4:**
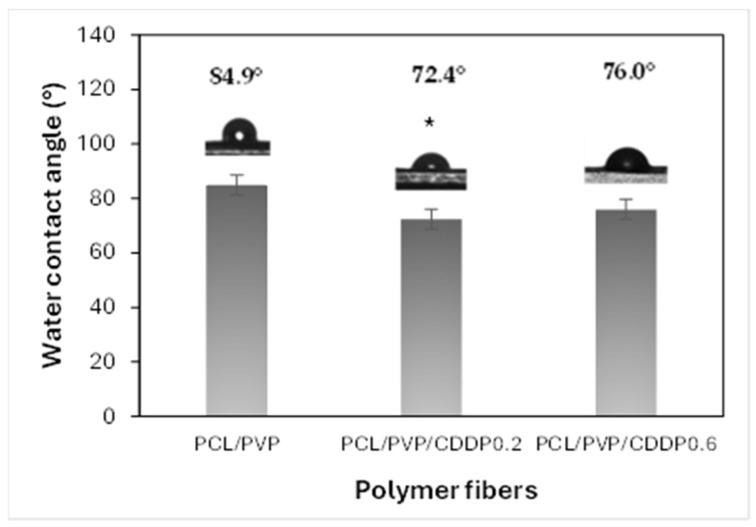
Water contact angle analysis of PCL/PVP and PCL/PVP/CDDP electrospun coaxial fibers. Statistical significance: * *p* < 0.05 when compared to PCL/PVP.

Contact angle measurement is an indicator of the degree of hydrophilicity/hydrophobicity, which can predict the wettability, adhesion, and dissolution capacity of fibers in the vaginal environment. In addition, wettability is of great importance for the subsequent diffusion of the drug carried by polymeric fibers [28,29]. The contact angle classification is divided into four categories, depending on the angle value measured. Surfaces with an angle < 25° and 25 to 90° are considered super hydrophilic and highly hydrophilic, respectively. On the other hand, if the measured surface has an angle of 90 to 150° or >150°, it is hydrophobic or superhydrophobic; the smaller the contact angle with water, the more hydrophilic the surface of the material. As shown in Figure 4, both fibers of coaxial fibers without CDDP and those loaded with the drug are highly hydrophilic, since the contact angle value is less than 90° [10]. Previous studies reported water contact angle values of 115 to 130° in PCL composite fibers, confirming their hydrophobic nature [8,15]. However, when combined with other polymers of a hydrophilic nature, such as chitosan or PVP, either in uniaxial or coaxial sheath/core fibers, the contact angle decreases, improving the hydrophilicity of the material surface. Although the fibers developed in this study comprise hydrophobic PCL, the hydrophilic PVP polymer favors the fiber’s hydrophilicity. The improved hydrophilicity caused by the action of PVP, which is not found on the fibers’ surface, may be due to low amounts of this polymer present in the sheath part; the fibers’ porosity and the relative permeability of PCL enable this filtration.

### 3.4. Mechanical Properties of Coaxial Fibers

**Figure 5:**
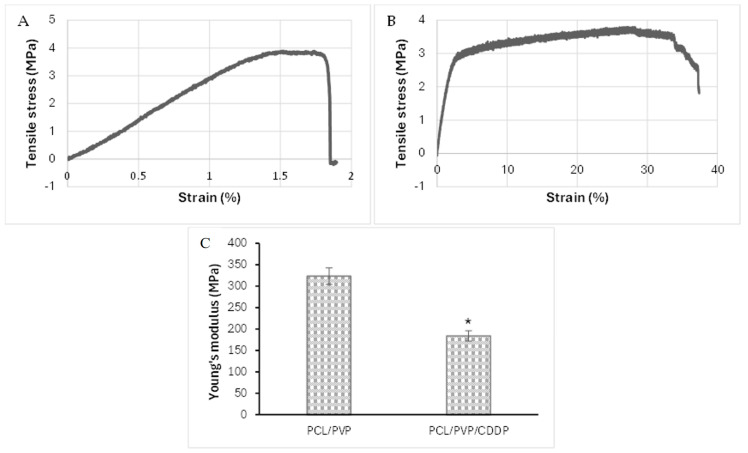
Mechanical properties of the electrospun coaxial fibers: (**A**) PCL/PVP stress–strain curve; (**B**) PCL/PVP/CDDP stress–strain curve; (**C**) Young’s modulus for the respective PCL/PVP and PCL/PVP/CDDP fibers. Statistical significance: * *p* < 0.05 indicated a statistically significant difference.

The mechanical strength of PCL/PVP and PCL/PVP/CDDP fibers was determined using the Instron universal machine. Figure 5 shows the stress–strain curves and Young’s modulus graph. PCL/PVP fibers presented tensile strength and strain of 3.88 MPa and 1.52 ± 0.27%, respectively. On the other hand, the fibers with incorporated CDDP showed similar tensile strength (3.80 MPa); however, the % strain was higher (27.12 ± 7.02%, 17 times higher). Similar results have been reported in previous studies where increased resistance to deformation was reported in nanofibers loaded with cisplatin and other drugs compared to the deformation in plain nanofibers [10,30]. Additionally, the results of Young’s modulus showed a noticeable difference between the fibers with CDDP and those without it, being higher in the latter (174.5 MPa vs. 309.6 MPa), which indicates that the fibers without pharmacological load present greater rigidity and resistance to deformation. However, as shown in the stress–strain curves, the PCL/PVP/CDDP fibers exhibit a high deformation capacity before breaking occurs; therefore, although it is less rigid, it may be a convenient option for vaginal application. Although it has been reported in previous works that the addition of PVP to PCL and other polymers in the manufacture of fibers compromises their mechanical properties due to poor interaction between polymers [31,32], in the PCL/PVP fibers developed in this study, the synergy between both polymers combined the solid mechanical resistance of PCL with the dissolving properties of PVP for pharmacological release [33,34,35].

### 3.5. Fiber Degradation

**Figure 6:**
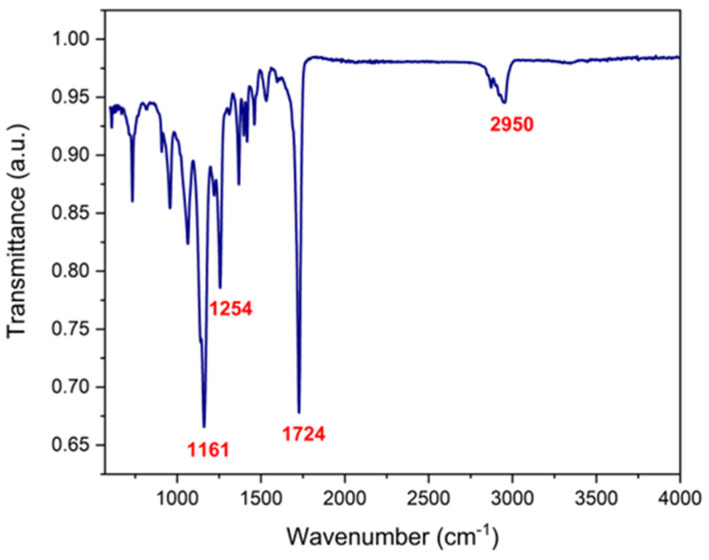
Evaluation of the degradability of coaxial PCL/PVP fibers: FTIR spectrum of the electrospun coaxial fibers at the end of the test.

The degradation of the coaxial fibers was determined by measuring their weight loss during different time intervals (Table 1). Notable weight loss occurred from day 10 in the fibers without cisplatin and in those containing it (51.4% and 45.2%); however, at the end of the test, the weight loss percentage did not vary significantly from that observed on day 10. These results demonstrate the biodegradability of the developed fibers. The pH of the test solution, which was initially low (4.07), similar to the acidic vaginal environment, did not show significant variations. Infrared spectra of the fiber fragments were obtained at the end of the test and it was determined that the weight loss occurred due to the degradation of the PVP polymer; in the spectrum (Figure 6), bands corresponding only to the PCL polymer are observed at positions 1161 cm^−1^ (C-O stretching), 1254 cm^−1^ (C-O-C stretching), 1724 cm^−1^ (C=O stretching), and 2950 cm^−1^ (C-H stretching); however, the bands corresponding to PVP do not appear, indicating that this last polymer is not present and has already been degraded. Previous studies have reported similar results in degradation assays of PCL and PVP polymers, finding that the latter dissolves rapidly. At the same time, PCL requires a longer time to degrade [36]. PCL has been reported to require extended periods for hydrolytic degradation [37]. Various working groups have reported low degradation rates in coaxial fibers containing PCL; additionally, they have determined that the weight loss was mainly due to other hydrophilic polymers that are part of the polymeric structure [38,39]. In our laboratory, the real-time degradation test, ISO 10993-13 standard [40] is being conducted, in which the exposure time is extended to determine the time of complete degradation of the fibers.

### 3.6. In Vitro Release of Cisplatin

**Figure 7:**
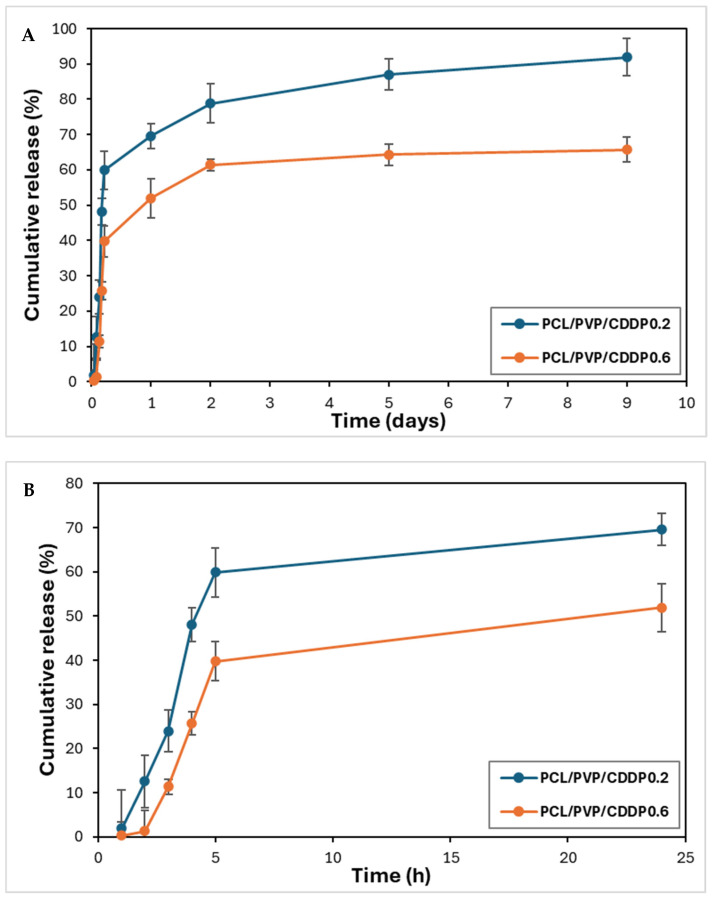
Cumulative release profile of cisplatin from PCL/PVP electrospun coaxial fibers incubated in 1X PBS, pH 4.07, 37 °C. (**A**) Cisplatin release graph at 9 days. (**B**) Cisplatin release graph at 24 h.

The use of micro/nanofibers as drug delivery systems by electrospinning provides the possibility of efficient encapsulation. The encapsulation efficiency was calculated (EE% = (Total amount of determined cisplatin/Initial amount of cisplatin)*100) by UV-Vis spectroscopy measurements at 202 nm after the complete dissolution of a fiber fragment in PBS. The total amount of determined cisplatin refers to the amount of free cisplatin in the supernatant, and the initial amount of cisplatin refers to the amount of the total amount of cisplatin added. The encapsulation efficiency of cisplatin was ~66–92%, which is consistent with similar values of % encapsulation of cisplatin and other bioactive substances in nanofibers reported in previous studies [10,14,30]. Data from the in vitro cisplatin release test from coaxial fibers are presented in Figure 7A,B. The samples showed a biphasic release profile: a rapid initial release followed by a slow and sustained release. The burst release of the first stage occurred in the first 5 h, reaching a CDDP release of 59.9% and 39.7%. After this initial release, the constant and sustained release of the drug occurred, finding a cumulative release on day 9 of 92% and 65.7% for the fibers with lower and higher cisplatin load, respectively; this two-stage release of CDDP and other bioactive substances as tissue regeneration promoters from polymeric nanofibers has already been reported by different working groups [17,41]. The release mechanism is probably diffusion through the manufactured fibers’ pores, holes, or imperfections. Because PVP is a hydrophilic polymer and therefore soluble in water, it dissolves, releasing CDDP (initial release phase) and, in turn, creates pores through which the buffer can penetrate to the innermost part of the coaxial fiber to achieve diffusion and release of the remaining CDDP during the second, slow and sustained phase. Other working groups have described similar results for releasing substances such as bioactive agents through devices composed of simple or coaxial polymeric fibers [42,43]. This hypothesis can be confirmed by the results of the degradation test, where the weight loss was approximately half of the initial weight and the FTIR spectrum shows the absence of the PVP polymer, indicating its dissolution.

Furthermore, according to the results obtained from the water contact angle measurements, the surface of the fibers is highly hydrophilic, since the values obtained were low; therefore, the surface (PCL coating) probably contains PVP in low quantities due to filtration from the nuclear part of the fiber, which contributes to the formation of cavities that facilitate drug diffusion. In turn, due to its hydrophobic nature, the presence of coaxial fibers of PCL polymer in the sheath reduces the burst release. Previous studies have reported that PCL can be a protective barrier to prevent rapid drug release in sheath/core structures [10,28]. According to the cisplatin release profile in our study, it is possible to use the coaxial fibers sheath/core PCL/PVP as a controlled-release delivery system.

### 3.7. In Vitro Biosafety Assays of the Coaxial Fibers

**Figure 8:**
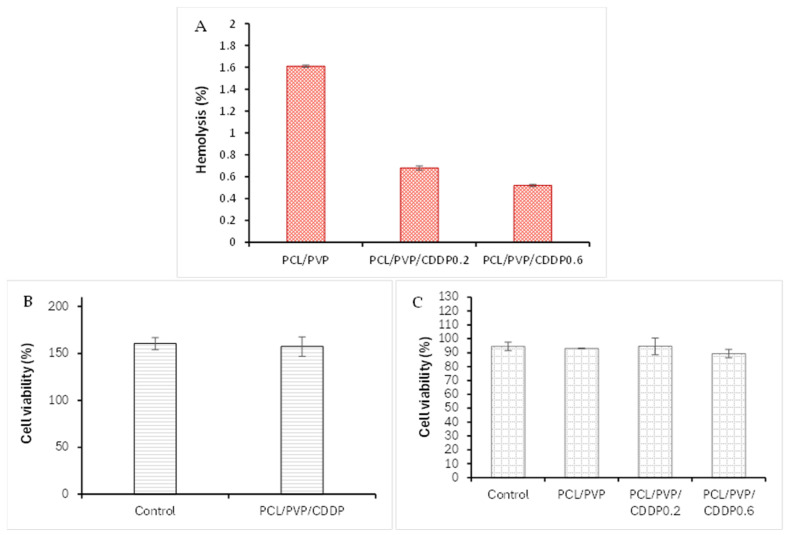
Cell assays with exposure to electrospun coaxial fibers: (**A**) hemocompatibility through measurement of % hemolysis of red blood cells when exposed to PCL/PVP and PCL/PVP/CDDP electrospun fibers; (**B**) % cell viability in NIH3T3 cells; and (**C**) % cell viability in HeLa cells.

The % hemolysis, biocompatibility, and cytotoxicity of the coaxial fibers were evaluated in erythrocytes, NIH3T3 cells, and HeLa cells, respectively; the graphs with the results are shown in Figure 8. The % hemolysis for PCL/PVP fibers without the drug was 1.61%, while for fibers loaded with 0.2 mg and 0.6 mg of cisplatin, the % hemolysis was 0.68% and 0.52%, respectively, Figure 8A. All fiber segments tested in the analysis presented % hemolysis less than 2%, which is lower than the accepted values (<5%) as proposed by ASTM F756-00 [44]. Therefore, PCL/PVP coaxial fibers can be used in localized cervical cancer therapy without causing hemolysis of red blood cells, since the very low hemolysis percentage indicates good blood compatibility of the biopolymers. The biocompatibility test results performed on NIH3T3 mouse fibroblasts showed no significant difference in cell viability between cells exposed to coaxial fibers and control cells, suggesting the biocompatibility of the biomaterials used (Figure 8B). Previous studies have reported the excellent compatibility of PCL polymer fibrous scaffolds in mouse dermal fibroblasts and human dermal fibroblasts [36,45,46,47].

On the other hand, the results obtained in the cytotoxicity assay in HeLa cells showed similar cell viability in cells exposed to PCL/PVP fibers without the drug and with control cells (93.01% vs. 94.45%). The viability was comparable when comparing cells exposed to fibers loaded with CDDP at a low concentration with control cells (94.47%). On the other hand, although there was no significant difference in cell viability between cells exposed to fibers loaded with cisplatin at a higher concentration compared to control cells, a slight decrease in cell viability was observed (89.33%), Figure 8C, which suggests either that a higher drug load is needed within the fibers or that the fibers contain the drug efficiently encapsulated in their PVP nuclear part and a longer exposure time to the fibers is necessary, since the cytotoxicity test was performed with a 24-h exposure. The encapsulation efficiency allows the developed fiber system to be used as a local drug carrier with controlled release, dependent on polymeric degradation.

### 3.8. Mucoadhesion and Residence Time

**Figure 9:**
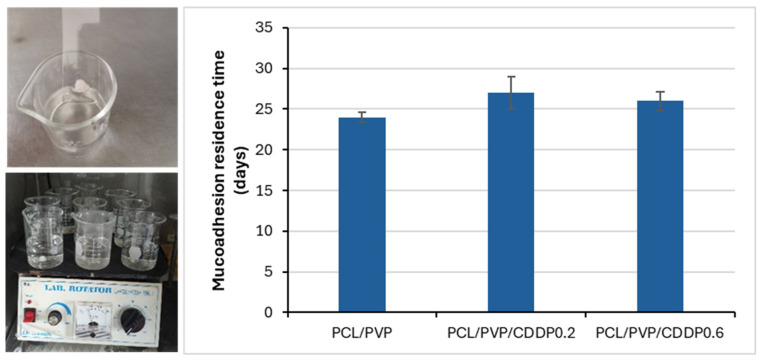
Mucoadhesion assay on rat vaginal tissue to determine the residence time of electrospun PCL/PVP, PCL/PVP/CDDP coaxial fibers.

Local therapy aims to administer cisplatin chemotherapy using the developed fibers through the vaginal route, which provides easy access to the cervix and is currently used for the application of bioactive substances through various pharmaceutical forms, such as ovules or gels. Therefore, the administration of cisplatin encapsulated in coaxial fibers will be done through direct intravaginal insertion, with medical assistance to position it at the target site. This will be done through a minimally invasive procedure like a Pap test. Due to the biocompatible nature of the polymers used in the developed coaxial fiber system, it is possible that it does not cause vaginal irritation. However, it is essential to evaluate the effects that the drug can have on the healthy vaginal mucosa adjacent to the target site of the therapy. The mucoadhesion test allowed us to evaluate the capacity of the fibers to adhere to the vaginal mucosa at the time of administration, since the objective is to maintain it for a prolonged period to allow the release of the drug in a sustained and controlled manner. The simulated vaginal fluid used in the test imitates the vaginal environment [48,49]. Adherence of the fiber fragments to the vaginal mucosa was observed for more than 15 days without evidence of detachment, despite being kept in constant agitation and FVS, Figure 9, both in the fibers without the drug and those containing it. These results allowed us to determine that the coaxial fibers present a high mucoadherence to the vaginal wall. To obtain an effective system for the localized release of chemotherapeutic agents at the vaginal level, adherence is a critical property, as it ensures that the fibers remain in place for a sufficient period for the release of the drug. The PVP polymer, a component of the electrospun fibers, provides adhesive properties that improve intravaginal mucoadherence [50,51]. Some factors can limit the application of intravaginal systems for the transport and action of drugs, such as vaginal exchange, which is the constant production and secretion of fluids that allow the elimination of harmful substances, a process that occurs every 96 h [52,53]; however, the fabricated electrospun nanofibers released the highest amount of cisplatin before turnover occurred, according to the results of the release assay, and the residence time of the fibers was longer than the turnover time.

## 4. Conclusions

The coaxial electrospinning technique was successfully employed to develop cisplatin-loaded polycaprolactone/polyvinylpyrrolidone sheath/core fibers. The fabricated fibers showed continuous, microsphere-free morphology, a well-defined sheath/core structure, and diameters ranging from 3 to 9 µm, indicating a promising drug microcarrier system. The microfibers demonstrated a two-phase cisplatin release profile: an initial burst release followed by a slow release over eight days. Polycaprolactone provided mechanical strength to the fibers, and due to its long hydrolytic degradation time and hydrophobic nature, it was possible to prevent the burst release of the entire encapsulated drug. On the other hand, polyvinylpyrrolidone provided hydrophilic and adhesive properties to the surface of the fibers, which favors dissolution in the vaginal environment and mucoadhesion of the fibers to the intravaginal wall for a sufficient time to allow the release and bioavailability of the drug at the target site. As demonstrated by erythrocyte and NIH3T3 cell exposure, coaxial fibers exhibited high cytocompatibility. The drug delivery system developed using microfibers could improve the efficacy of cisplatin treatment through local vaginal application. With local administration of the chemotherapeutic agent, it would be possible to reduce the dose and side effects of cisplatin when administered systemically, considerably improving the patient’s quality of life. Currently, various types of devices are used for vaginal drug administration, such as films, gels, and vaginal tablets; however, their effectiveness is diminished by disadvantages such as low drug loading capacity, limited vaginal retention, or rapid pharmacological release. The coaxial electrospun fibers developed in this work present release of the chemotherapeutic for a more extended period than that reported in other pharmaceutical forms such as films; in addition, the adhesion time to the vaginal mucosa is longer when compared to other devices such as gels, vaginal tablets, or ovules, allowing the fibrous system to remain long enough for the release of the drug. Based on the results, the electrospun fibers present desirable characteristics for drug delivery in treating cervical cancer; however, ex vivo and in vivo studies are essential before their use in patients. Using polymer combinations with different properties and the electrospinning technique with its modifiable parameters allow the fabrication of nano- and microstructures with improved characteristics, such as mucoadhesiveness and optimal release profiles, for their application in treating this complex disease.

## Figures and Tables

**Table 1:** Weight loss due to degradation of PCL/PVP and PCL/PVP/CDDP fibers.

	% Weight Loss				
	10 Days	14 Days	1 Month	3 Months	6 Months
PCL/PVP fibers	51.4 ± 0.67	51.6 ± 0.64	51.6 ± 0.62	51.6 ± 0.61	51.6 ± 0.67
PCL/PVP/CDDP fibers	45.2 ± 0.26	45.6 ± 0.38	46.1 ± 0.40	46.2 ± 0.35	46.2 ± 0.38

## Data Availability

Specific data of this study may be available upon request from the corresponding author.

## Supplementary Materials

The following supporting information can be downloaded at: https://www.mdpi.com/article/10.3390/polym17050637/s1, Table S1. Images of uniaxial fibers with different PCL concentrations, voltage, and flow rate. All scale bars are 20 and 100 μm; Table S2. Images of uniaxial fibers with different PVP concentrations, voltage, and flow rate. All scale bars are 20 and 100 μm; Table S3. Images of coaxial fibers with different concentrations of PCL and PVP, voltage, and flow rates. All scale bars are 20 μm; The graphical abstract was created in BioRender, https://BioRender.com/b68b902. It is licensed under an Academic Publishing License.
