# Supplementary material for: Electrospun Coaxial Polycaprolactone/Polyvinylpyrrolidone Fibers Containing Cisplatin: A Potential Local Chemotherapy Delivery System for Cervical Cancer Treatment
Source: Polymers (Basel). 2025 Feb 27;17(5):637. doi: 10.3390/polym17050637 (PMC11902410; doi:10.3390/polym17050637)
Supplement: Supplementary file 1 [file polymers-17-00637-s001.zip › polymers-3457516-supplementary.pdf]

polymers

MDPI

Supplementary Material

Electrospun Coaxial Polycaprolactone/Polyvinylpyrrolidone Fibers Containing Cisplatin: A Potential Local Chemotherapy Delivery System for Cervical Cancer Treatment

Mariana Sarai Silva-Lopez [1], Vladimir Alonso Escobar-Barrios [2] and Luz Eugenia Alcantara-Quintana [1,*]

[1] Coordination for the Innovation and Application of Science and Technology (CIACYT), Autonomous University of San Luis Potosi, 550-2a Sierra Leona Ave, San Luis Potosi 78210, Mexico; marianasillop@gmail.com
[2] Advanced Materials Department, Institute for Scientific and Technological Research of San Luis Potosi A.C. Road to San Jose Dam, Lomas 4a Section, San Luis Potosi 78216, Mexico; vladimir.escobar@ipicyt.edu.mx
* Correspondence: luz.alcantara@uaslp.mx

The optimal process conditions were initially determined for the fibers separately to carry out coaxial electrospinning and obtain continuous fibers without beads or interrupted segments, that is, for uniaxial polycaprolactone fibers and uniaxial polyvinylpyrrolidone fibers. The coaxial fibers were then optimized, for which the parameters obtained in single electrospinning were essential. The modified process parameters were the polymer solution's internal and external flow rate, voltage, and concentration-viscosity. The distance between the needle and the collector and the collector's rotation speed were set at 10 cm and 230-290 rps.

Table S1. Images of uniaxial fibers with different PCL concentrations, voltage, and flow rate. All scale bars are 20 and 100 μm.

Experiment	PCL Conc. (w/v%)	Voltage (kV)	Flow rate (mL/h)	Image
E1	18	8	3	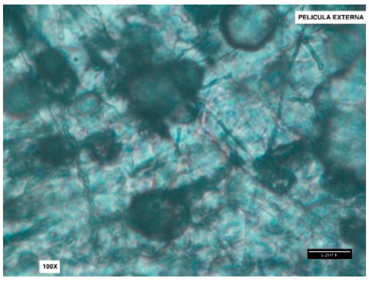
*E2	25	8	3	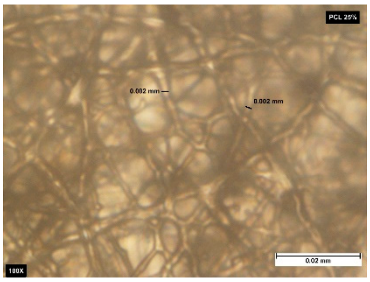

E3	25	8	3.5	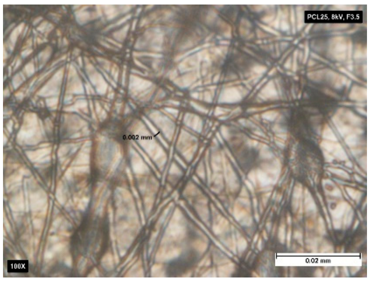
E4	25	8	4	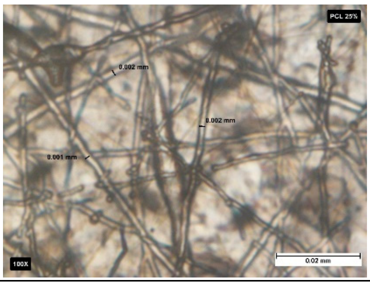
E5	25	8	5	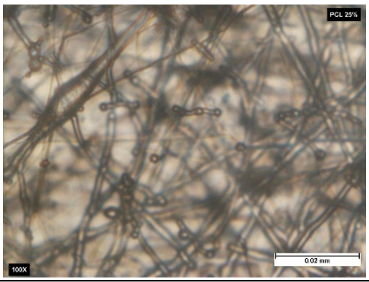
E6	25	9	3	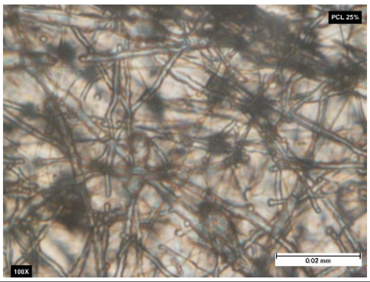
E7	25	10	3	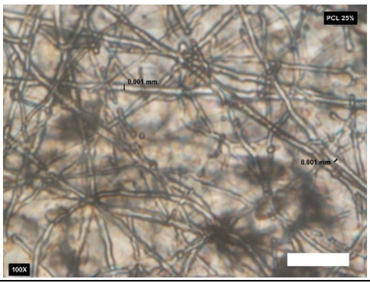

*Optimal parameters chosen

Table S2. Images of uniaxial fibers with different PVP concentrations, voltage, and flow rate. All scale bars are 20 and 100 μm.

Experiment	PVP Conc. (w/v%)	Voltage (kV)	Flow rate (mL/h)	Image
E1	60	8.5	1	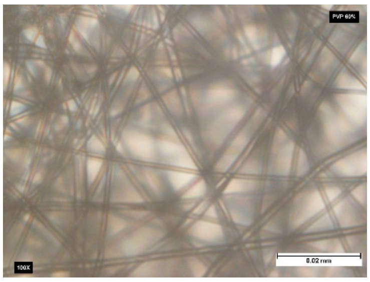
E2	80	7	1	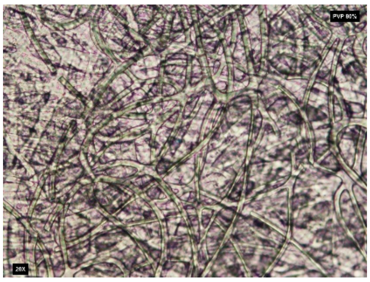
*E3	80	8	2	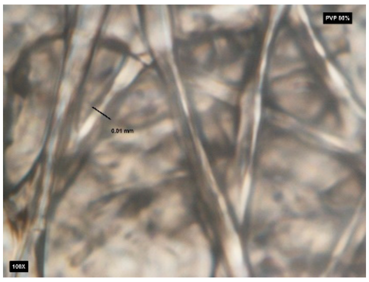
E4	80	8	3	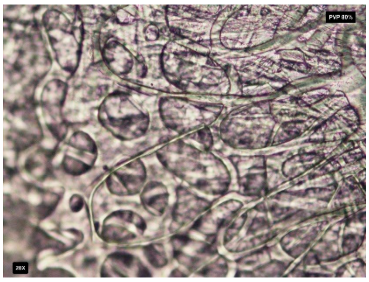
E5	80	8	3.5	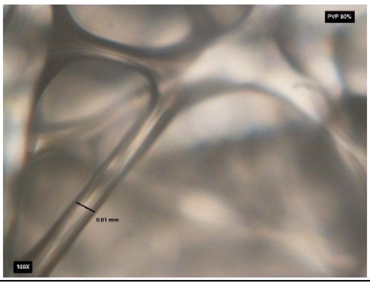

E6	80	8	4	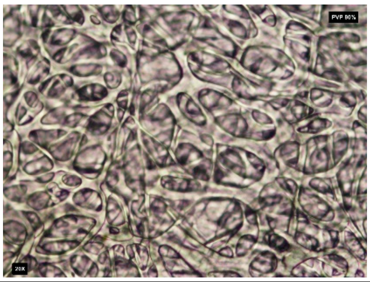
E7	80	9	3	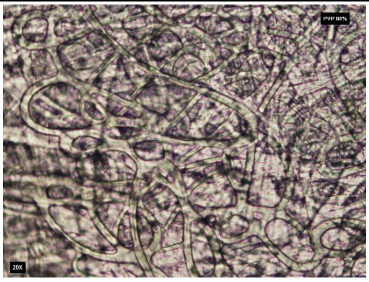

*Optimal parameters chosen

Table S3. Images of coaxial fibers with different concentrations of PCL and PVP, voltage, and flow rates. All scale bars are 20 µm.

Experiment	PCL Conc. (w/v%)	PVP Conc. (w/v%)	External Flow Rate (mL/h)	Internal Flow Rate (mL/h)	Voltage (kV)	Image
E1	30	80	3	2	13	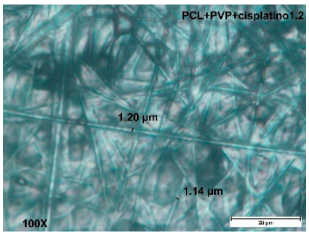
*E2	45	85	3	2	++13-16	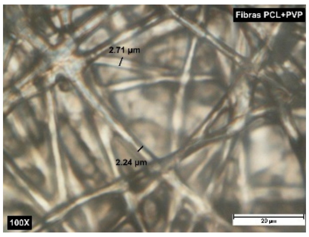

*Optimal parameters chosen

++ The applied voltage was variable in forming coaxial fibers with the different cisplatin concentrations (0.2 and 0.6 mg/mL); an increase in voltage was necessary to form fibers with the highest drug concentration.
